# Puerarin Increases Survival and Protects Against Organ Injury by Suppressing NF-κB/JNK Signaling in Experimental Sepsis

**DOI:** 10.3389/fphar.2020.00560

**Published:** 2020-05-07

**Authors:** Lei Wang, Qiao Liang, Anqi Lin, Xiufang Chen, Yongzhen Wu, Bin Zhang, Yu Zhang, Haiyan Min, Yanting Wen, Shiyu Song, Qian Gao

**Affiliations:** ^1^Center for Translational Medicine and Jiangsu Key Laboratory of Molecular Medicine, Medical School of Nanjing University, Nanjing, China; ^2^Department of Biochemistry, School of Basic Medical Sciences, Wenzhou Medical University, Wenzhou, China; ^3^Central Laboratory, Nanjing Chest Hospital, Medical School of Southeast University, Nanjing, China

**Keywords:** puerarin, lipopolysaccharides, sepsis, cecal ligation and puncture, liver injury, macrophage

## Abstract

Puerarin, an isoflavonoid rich in Radix *Puerariae*, has been reported to be a broadly effective regulator in various biological processes and clinic conditions. However, the role of puerarin in sepsis-induced mortality with multiple-organ injury remains unknown. Herein, we showed that puerarin potently attenuated organ injury and increased survival rate in both lipopolysaccharides (LPS) and cecal ligation and puncture (CLP) induced mouse sepsis models. It greatly suppressed systemic inflammation, determined by the serum levels of proinflammatory factors TNF-α, IL-6, IL-1β, IL-10, as well as monocyte chemotactic protein-1 (MCP-1) and C-reactive protein (CRP). Flow cytometry analysis indicated that puerarin settled overall inflammation mainly by normalizing expanded macrophages with limited effects on dendritic cells and CD4^+^T cells in the circulation of sepsis mice. In the liver, puerarin inhibited the transcription of inflammatory factor TNF-α, IL-6, and IL-1β and protected hepatocyte apoptosis in sepsis mouse models. In vitro, puerarin inhibited LPS-induced inflammation in LO2 hepatocytes, prevented TNF-α-mediated cell apoptosis and promoted an M2 phenotype revealed by M2 marker IL-10 and Arginase-1 (Arg-1) in LPS challenged Raw 264.7 macrophages, through the inhibition of TLR4/NF-κB/JNK pathway. In conclusion, puerarin reduced systemic inflammation and protected organ injury in sepsis mice, thus, it might provide a new modality for a better treatment of sepsis.

## Introduction

Sepsis is a life-threatening condition that arises when the systemic response to infection injures its own tissues and organs (Angus and van der Poll, 2013). It is a common complication after severe trauma and major surgery and is the leading cause of death in critically ill patients (Martin et al., 2003). Although the pathologic mechanism responsible for sepsis is still incomplete, it is believed that an altered host immune function that leads to a harmful cytokine storm and a compromised antibacterial immunity, resulting in the susceptibility to secondary infection, plays a key role in the pathogenesis of organ failure (Hotchkiss et al., 2013; Iskander et al., 2013).

The liver is the most commonly injured organs during sepsis. Its dysfunction is one of the key hallmarks of multiple-organ failure during the development of sepsis (Yan et al., 2014). An acute liver injury may occur at any stage of sepsis (Canabal and Kramer, 2008) and the patients of sepsis are usually predicted with abnormal liver function exacerbations (Ashare et al., 2006). Moreover, liver damage caused by sepsis may not only lead to liver failure but also aggravate the overall condition of the patients, directly affect its prognosis, and death (Kramer et al., 2007). Therefore, an early therapeutic application of liver protection may significantly improve the prognosis of sepsis. Currently, the clinical treatment of sepsis is still heavily relying on broad-spectrum antibiotics and nutritional support; however, the mortality rate has not been significantly reduced in the past 20 years (Shapiro et al., 2007; Wenzel and Edmond, 2015). In addition, overuse of antibiotics has caused serious public health concerns (Russell, 2006). Thus, there is a clinically urgent need for novel and effective approaches for sepsis.

Puerarin (PUE) has been observed to be useful in various conditions (Zhou et al., 2014), including fever, diarrhea, emesis, toxicosis, cardiac dysfunctions, and metabolic disorders (Zhang et al., 2013). It was suggested to possess multiple biological activities, including antioxidant, anti-carcinogenic functions (Wong et al., 2011), and inhibiting LPS-induced NF-κB signaling in macrophages (Hu et al., 2011). Despite a long history of its usage, there is no study of puerarin in sepsis.

In the present study, the therapeutic effect of PUE on animal mortality and organ injuries, particularly in the liver, were evaluated in both LPS- and CLP-induced mouse sepsis models. The purpose of using the two sepsis models was to determine whether LPS mimics gut bacteria-originated sepsis which is clinically more relevant and to better conclude puerarin's clinic potential. Importantly, LPS directly targets TLR4 signaling. In addition, the protective effect of puerarin on hepatocytes and its anti-inflammatory effect on macrophages were also investigated *in vitro*. The underling mechanisms of puerarin effect on cells were explored.

## Materials and Methods

### Reagents

LPS (*Escherichia coli* 055: B5) and puerarin were purchased from Sigma Chemical Co. (St. Louis, USA). rmTNF-α and enzyme-linked immunosorbent assay (ELISA) kits for cytokines were obtained from R&D (Minneapolis, USA). Enzyme activities of alanine aminotransferase (ALT), aspartate aminotransferase (AST), superoxide dismutase (SOD), and malondialdehyde (MDA) detection kits were from Beyotime Co. Ltd. (Shanghai, China). Antibodies used for Western blot were all purchased from Cell Signaling Technology (Danvers, USA).

### Animal Procedure

Animal welfare and experimental design were carried out strictly in accordance with the Guide for the Care and Use of Laboratory Animals (Nanjing University, ethical approval number: IACUC-2003071), and the procedure was conducted strictly in accordance with the “Guidelines for Experimental Animals” of the Ministry of Science and Technology (2006, Beijing, China).

Specific pathogen-free (SPF) male C57BL/6J mice (6-week-old) were obtained from Model Animal Genetics Research Center of Nanjing University (Nanjing, China). All mice were housed in SPF condition with a 12:12 h light-dark cycle, free access to water and food.

For LPS-induced sepsis model, the mice were intraperitoneally injected (i.p.) with LPS (20 mg/kg), PBS as control. The animals were randomly divided into control group, LPS group, and LPS+PUE (160 mg/kg) group.

For CLP-induced model, the mice (CLP group) received fecal peritonitis according to a previously reported protocol (Rittirsch et al., 2009). Briefly, to induce a mid-grade sepsis, the cecum of the mouse was exposed and ligated at half the distance between distal pole and the base of the cecum, then, punctured through from mesenteric toward antimesenteric direction after ligation. The mice in control group received a sham operation, while the cecum was exposed without ligation and puncture. The CLP+PUE group mice received puerarin (160 mg/kg) intraperitoneally after CLP operation.

**Figure 1:**
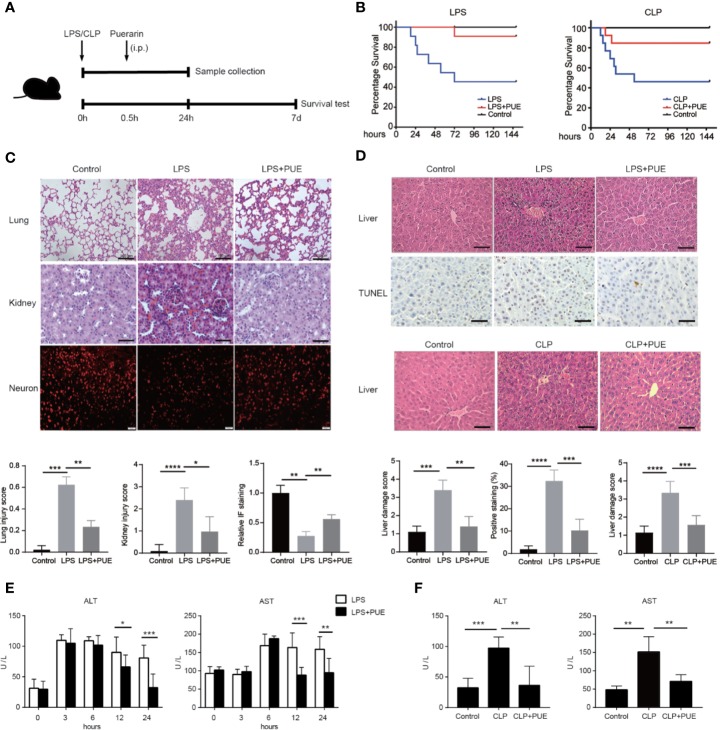
Puerarin increased overall survival and protected multiple-organ failure in sepsis mice. **(A)** A sketch of the experiment was illustrated. Mice were treated with puerarin (160 mg/kg, intraperitoneal injection) 30 min after LPS exposure or CLP operation. The mice for tissue collection were sacrificed 24 h after the challenge. For survival analyze, a 7-day follow-up was performed **(B)** The KM survival curve were plotted to demonstrated survival condition of both LPS and CLP mice models (n = 10). **(C)** Upper panel: H&E staining of lungs and kidneys of sepsis mice, scale bar 100 μm. Immunofluorescence staining of neurons by NeuN antibody in the brains of sepsis mice, scale bar 50 μm. Lower panel: the quantification of the indicated scores of each staining (n = 6) **(D)** Top panel: H&E staining and terminal deoxynucleotidyl transferase-mediated dUTP nick end labeling (TUNEL) staining of the liver sections in LPS sepsis mouse model. The apoptotic cells showed a dark-brown nucleus, scale bar 100 μm. Middle panel: H&E staining of the liver sections in CLP mouse model, scale bar 100 μm. Bottom panel: quantification of indicated scores of each staining in the upper panels (n = 6). **(E)** Enzyme activities of serum alanine aminotransferase (ALT) and aspartate aminotransferase (AST) were analyzed at indicated time points in LPS sepsis mouse model (n = 5). **(F)** Enzyme activities of serum ALT and AST were analyzed at 24 h after the CLP operation in CLP sepsis mouse model (n = 5). Data were expressed as mean ± SD, *P < 0.05, **P < 0.01, ***P < 0.001, ****P < 0.0001.

For survival study, the animals of all groups (n = 10) were monitored for 7 days. The specific number of mice used in each experiment was indicated in the figure legends. For initial blood drawing: n = 10, for tissue collection: n = 8 (**Figure 1A**).

The blood samples of the mice were collected from tail vein at time point of 0, 3, 6, 12, and 24 h after the injection of LPS, and 24 h after the CLP operation. The mice were sacrificed at 24 h for tissue collection or at day 7 for survival studies by cervical dislocation.

### Cytokine and Liver Enzyme Detection

The serum concentrations of TNF-α, IL-6, IL-1β, and IL-10, as well as MCP-1 (Monocyte chemotactic protein 1) and C-reactive protein (CRP) were determined using ELISA kits according to the manufacturer's instructions. The plasma enzyme activities of ALT and AST were determined using ALT and AST detection kits according to the manufacturer's instructions.

### Histology, Immunohistochemistry, and Immunofluorescence Analyses

The brain, liver, lung, and kidney tissues were obtained after the cervical dislocation of experimental mice, the tissues were fixed in 4% paraformaldehyde and embedded in paraffin. The sections (~10 μm) of various organs were stained with hematoxylin & eosin (H&E) for conventional morphological evaluation under light microscope (Olympus, Tokyo, Japan). Briefly, the lung injury scores were according to a recently published criteria, considering neutrophils in the alveolar space (A) and in the interstitial space (B), hyaline membranes (C), proteinaceous debris filling the airspaces (D), and alveolar septal thickening (E). Score = [(20 × A) + (14 × B) + (7 × C) + (7 × D) + (2 × E)]/(number of fields × 100) (Matute-Bello et al., 2011). The kidney injuries were scored using a semiquantitative scale designed to evaluate the degree of necrosis, cell loss, and necrotic casts on a five-point scale based on extent of involvement as follows: 0, normal kidney; 0.5, 10%; 1, 10% to 25%; 2, 25% to 50%; 3, 50% to 75%; and 4, 75% to 100% (Ascon et al., 2009). Liver injury was defined as the amount of destruction of hepatic lobules, infiltration of inflammatory cells, hemorrhage, and hepatocyte necrosis, and estimated according to the following criteria: 1, 0% to 25% of damage; 2, 25% to 50% of damage; 3, 50% to 75% of damage; 4, 75% to 100% of damage (Baranova et al., 2016). All evaluations were quantified by two investigators blinded to the treatment.

For the immunohistochemistry analysis, the slides were blocked with goat serum and incubated with primary antibody (rabbit anti-F4/80) after antigen retrieval, overnight at 4°C. An Elite ABC kit and DAB substrate was used for the immunohistochemistry analysis. For the immunofluorescence analysis, 4% paraformaldehyde fixed brain tissues were embedded in paraffin, and sectioned into 7-μm slices. The slides were blocked with goat serum and incubated with primary anti-NeuN antibody (ABN78, USA) overnight at 4°C. Alexa Fluor 594 (Invitrogen, USA) was used as the secondary antibody. The brain immunofluorescence (IF) images were captured by a confocal microscopy (Olympus, Japan). The intensity and area of immunofluorescence staining was quantified by Fiji Software (Schindelin et al., 2012). The IF staining score was calculated by multiply average light intensity with area of immunofluorescence staining. The immunohistochemistry photos were color-separated by color deconvolution using the H-DAB method. The optical density and the area of DAB staining of color-separated picture were calculated by adjusted threshold in Fiji Software. The level of expression was acquired by multiplying optical density with area of DAB staining. The results were normalized to control to generate the relative expression of the staining.

### Terminal Deoxynucleotidyl Transferase-Mediated dUTP Nick End Labeling (TUNEL)

The liver apoptosis was assayed using In Situ Cell Death Detection Kit from Roche (Indianapolis, USA) according to the manufacturer's instructions. The terminal transferase reactions finally produced a dark-brown precipitate. The sections were counterstained slightly with hematoxylin. The percentage of positive stating cells was calculated by “analyze particle” method using Fiji software.

### Cell Culture

Human liver cell line LO2 and Murine macrophage cell line RAW 264.7 were purchased from Type Culture Collection of the Chinese Academy of Sciences (Shanghai, China). The cells were grown in Dulbecco's modified Eagle's medium (DMEM) supplemented with 10% (v/v) fetal bovine serum (FBS) and 1% penicillin streptomycin (Gibco, USA) in humidified incubators (Thermo, USA) at 37℃ under 5% CO_2_. Cell models of liver injury (LO2 cells) and macrophage activation (RAW264.7 cells) in sepsis were established by seeding LO2 or RAW264.7 cells in 6-well plates, respectively. The cells were treated with LPS or LPS+PUE (the concentrations for LPS and puerarin in each experiment concerned see figure legend) for 24 h, and lysed for western blot analyses. For viability test, the cells were seeded at a density of 1 × 10^5^ cells/mL in 96-well plates with four replications and starved for 24 h without serum before challenge, and the cell viability was analyzed by CellTiter 96^®^ AQueous One Solution Cell Proliferation Assay (MTS) kit (Promega, USA).

### Protein Extraction and Western Blot

Tissue or cell total proteins were extracted using Radioimmunoprecipitation assay (RIPA) buffer containing 1% SDS and the protein concentrations were determined by a BCA kit (Thermo, USA). There was 50 μg of total lysates separated by a 10% sodium dodecyl sulfate–polyacrylamide gel electrophoresis (SDS-PAGE) gel and transferred onto a Polyvinylidene fluoride (PVDF) membrane, and blocked with 5% bovine serum albumin (BSA) in Tris-buffered saline (TBS) for 90 min, and then incubated with appropriate primary antibodies over night at 4°C. After washing, the membrane was incubated secondary antibody for 90 min at room temperature, and visualized by using the ECL Plus western blotting detection reagents (Millipore, USA). The protein levels of Caspase-3, Cleaved caspase-3, TLR4, JNK, p-JNK, p65, p-p65, IκBα, TNFR1, Bas, Bcl-2, MyD88 were determined by Western blot assay. β-actin was used as an internal control.

### Quantitative Real-Time Polymerase Chain Reaction (qPCR)

Total RNAs were isolated from tissue samples and cells, using Trizol reagent and quantified. First-strand complementary DNA (cDNA) was synthesized using iScript cDNA Synthesis Kit (Vazyme, China). Quantitative PCR was performed with SYBR green PCR Master Mix (Vazyme, China) using viia 7 Real-Time PCR System (Applied Biosystems, CA). The primers were detailed in **Table 1**. The following cycle parameters were used: 55°C for 2 min, 95°C for 10 min, and 40 cycles of 95°C for 30 s, 60°C for 30 s. The relative expression of the target genes against that of the reference gene β-actin was calculated by 2^−ΔΔCT^ method. Samples were performed in triplicate and every experiment was performed at least three times. The transcription levels of IL-6, TNF-α, IL-1β, IL-10, iNOS, and Arg-1 were determined by qPCR assay.

**Table 1:** Primers used for Real-Time Quantitative PCR Analysis.

Gene	Forward primer	Reverse primer
β-actin	GGACGTACAACTGGTATTGTGC	TCGGCAGTAGTCACGAAGGA
IL-10	AGCCTTATCGGAAATGATCCAGT	GGCCTTGTAGACACCTTGGT
TNF-α	CAGGCGGTGCCTATGTCTC	CGATCACCCCGAAGTTCAGTAG
IL-6	TAGTCCTTCCTACCCCAATTTCC	TTGGTCCTTAGCCACTCCTTC
IL-1β	GCAACTGTTCCTGAACTCAACT	ATCTTTTGGGGTCCGTCAACT
iNOS	GTTCTCAGCCCAACAATACAAGA	GTGGACGGGTCGATGTCAC
Arg-1	CTCCAAGCCAAAGTCCTTAGAG	AGGAGCTGTCATTAGGGACATC

### Flow Cytometry

Single-cell suspensions of blood samples were washed with ice-cold PBS for three times and subsequently treated with FCM lysing solution for 15 min. Then, cells were washed with ice-cold PBS to remove the excess FCM lysing solution and stained with PE-anti-F4/80, FITC-anti-CD45, FITC-anti-CD3, APC-anti-CD11c, and PE-anti-CD4 antibodies from BD Bioscience (San Diego, USA) in the dark at RT for 15 min. The data were analyzed with FlowJo software (San Carlos, CA). The portion of mononuclear macrophage (CD45^+^, F4/80^+^), T helper cells (CD3^+^, CD4^+^) and dendritic cells (CD45^+^, CD11c^+^) were determined by flow cytometry.

### Data Analysis

Normally distributed data was analyzed by Student's t-test (for comparisons of two groups) or analysis of variance (for multiple group comparisons). For values that were not normally distributed (as determined by the Kolmogrov–Smirnov test), the Mann–Whitney's rank sum test was used. All statistical tests were two-sided with *P* < 0.05 considered statistically significant. Data is expressed as the Mean ± SD (standard deviation) and presented with GraphPad Prism 5 software (LaJolla, CA).

## Results

### Puerarin Increased Overall Survival and Protected Multiple-Organ Injuries in Sepsis Mice

A 60% of death rate was initially observed with the peak of death in the first 24 h in both LPS- and a mid-grade CLP-induced sepsis mouse models (median survival time: 63 h in LPS, 29 h in CLP). The treatment of puerarin significantly promoted survival and statistically doubled the survival rate (from 40% to 80% or higher) in both sepsis mice models (**Figure 1B**). In LPS-induced sepsis mice, puerarin significantly ameliorated edema, inflammatory cell infiltration, and severe hemorrhage in the lung and kidney (**Figure 1C**). In the brain, it protected the cortical neurons from endotoxin and significantly increased the staining of neuronal nuclei marker NeuN (**Figure 1C**). Importantly, in the liver, puerarin greatly reversed LPS as well as CLP-induced destruction of hepatic architecture and congestion and reduced broadly distributed cell death (**Figure 1D**). In consistent, puerarin decreased the serum levels of ALT and aspartate transaminase (AST), indicating a reduced liver damage in both models (**Figures 1E, F**). Thus, puerarin increased overall survival and protected multiple-organ injuries in sepsis.

### Puerarin Suppressed Systemic Inflammation and Normalized Expanded Macrophages in Sepsis Mice

**Figure 2:**
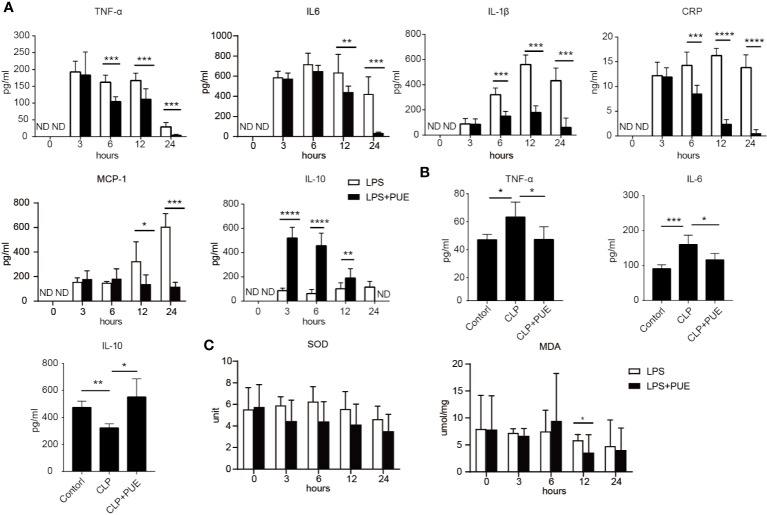
Puerarin suppressed systemic inflammation in sepsis mouse models. Blood samples of sepsis mice were harvested at 0, 3, 6, 12, and 24 h after LPS exposure in LPS sepsis model or at 24 h in CLP sepsis model. **(A)** The serum levels of TNF-α, IL-6, IL-1β, and CPR at indicated time points in LPS model were determined by ELISA (n = 4). **(B)** Serum levels of TNF-α, IL-6, and IL10 at 24 h after CLP operation were tested by ELISA in CLP mouse model (n = 4). **(C)** The levels of activity of serum SOD and MDA were tested at indicated time points in LPS model (n = 5). Data were expressed as mean ± SD. ND, not detected. *P < 0.05, **P < 0.01, ***P < 0.001, ****P < 0.0001.

We then examined the serum levels of inflammatory cytokine TNF-α, IL-6, IL-1β, and IL-10, and the clinical markers of acute systemic inflammation MCP-1 and CRP by ELISA in LPS-induced sepsis model. The inflammatory cytokines and factors tested were all significantly induced by LPS and inhibited by puerarin in sepsis mice (**Figure 2A**). Moreover, puerarin strongly increased the serum level of IL-10. Similar observations were made in CLP sepsis model (**Figure 2B**). Interestingly, neither LPS challenge nor puerarin treatment significantly altered the levels of two critical system oxidative stress markers, the key anti-oxidation enzyme SOD and the lipid peroxidation product MDA in sepsis mice, suggesting a systemic oxidative condition was not developed in sepsis (**Figure 2C**).

**Figure 3:**
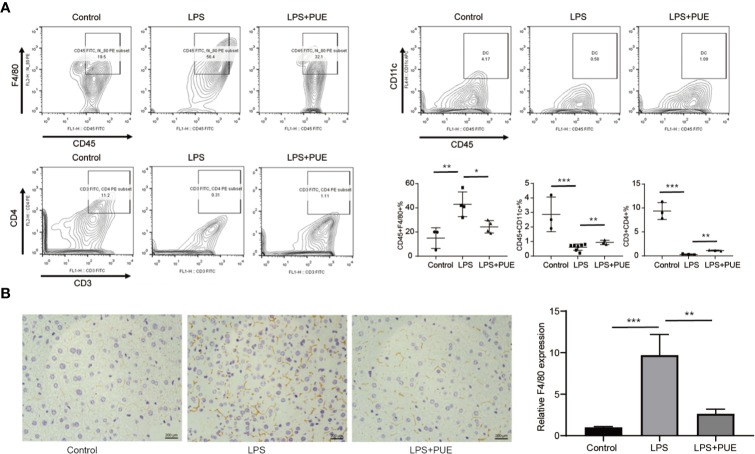
Puerarin suppressed periphery macrophage expansion and liver recruitment in sepsis mouse models. **(A)** The flow cytometry plots and analyses of the percentages of F4/80^+^ mononuclear macrophages, CD11c^+^ DCs and CD3^+^CD4^+^T cells in single-cell suspensions from the peripheral blood of mice 24 h after LPS treatment. Lower right panel shown the statistical results of indicated gates (n = 3 to n = 7). **(B)** Immunohistochemistry staining of F4/80 of the liver indicating recruitment of macrophage in the liver. Scale bar 200 μm. Right panel, the statistical result of quantification of the staining (n = 6). Data were expressed as mean ± SD, *P < 0.05, **P < 0.01, ***P <0.001.

Next, we analyzed the composition of immune cells in the circulation of sepsis mice. We found that the proportion of CD45^+^F4/80^+^ macrophages was greatly increased, whereas CD45^+^CD11c^+^ dendritic cells (DCs) and CD3^+^CD4^+^ T helper cells were dramatically decreased in LPS-induced sepsis mice when compared to those of controls. Noted of, while puerarin significantly restrained the expansion of macrophages, its effect on restoring DCs and CD4^+^ T cells in the peripheral blood in sepsis mice was rather limited (**Figure 3A**). Moreover, the large enrichment of macrophages in the liver of sepsis mice was also greatly suppressed by puerarin (**Figure 3B**). Thus, puerarin improved local and systemic inflammation in sepsis mice.

### Protection of Hepatocytes by Puerarin Involving Suppression of TLR4 and TNF-α Signaling Pathway

**Figure 4:**
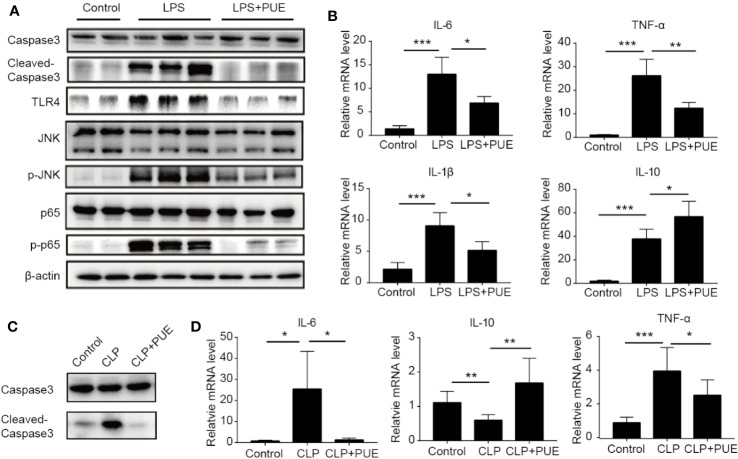
Puerarin inhibited liver inflammation by suppression of TLR4 signaling. **(A)** Western blot analyses of indicated proteins of the livers of mice. **(B)** The mRNA levels of IL-6, TNF-α, IL-1β, and IL-10 in the livers of mice were tested by real-time quantitative PCR (n=4). **(C)** Western blot analyses of caspase-3 and cleaved-caspase-3 in the livers of CLP model. **(D)** The mRNA levels of IL-6, IL-10, and TNF-α in the livers were tested by real-time quantitative PCR in CLP model (n = 4). Data were expressed as mean ± SD, *P < 0.05, **P < 0.01, ***P < 0.001.

We next studied the molecular nature of organ protection by puerarin in the liver. We found that puerarin strongly suppressed Caspase-3 cleavage (**Figure 4A**) in the liver of LPS induced sepsis mice, indicating a suppression of apoptosis. It inhibited LPS-induced TLR4 expression and the activation of its downstream NF-κB (p65) and JNK signaling. Furthermore, it downregulated the transcriptions of proinflammatory cytokine IL-6, TNF-α, and Il-1β and upregulated IL-10 transcription in the liver of sepsis mice (**Figure 4B**). Similar results were obtained in the CLP-induced sepsis mice (**Figures 4C, D**).

**Figure 5:**
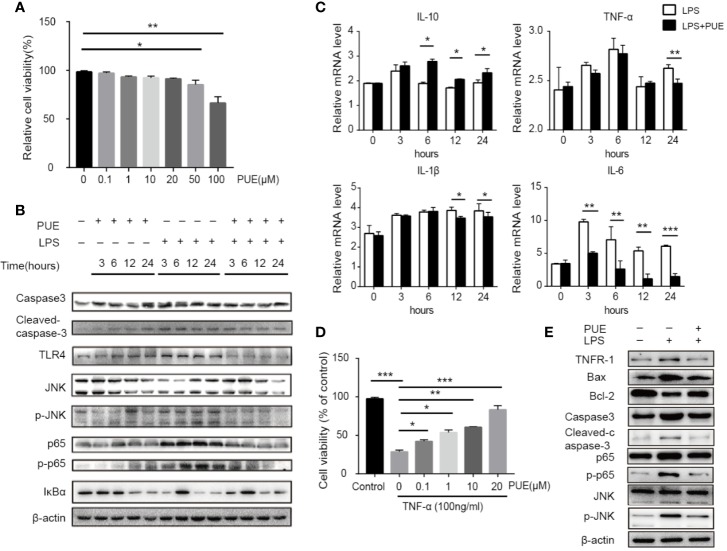
Puerarin inhibited LPS-induced inflammation and TNF-α-induced apoptosis by suppressing NF-κB and JNK signaling in hepatocyte. **(A)** LO2 cells were treated with puerarin at indicated concentrations for 24 h. The cell viability was determined by MTS assay (n = 5). **(B)** LO2 cells received indicated treatment of puerarin (20 μM) and LPS (100 ng/ml) for 3, 6, 12, and 24 h. Western blot analyses of indicated proteins in LO2 cells at different time points. **(C)** The mRNA levels of IL-10, TNF-α, IL-1β, and IL-6 of LO2 cells treated with LPS and puerarin for indicated time were tested by real-time quantitative PCR (n = 4). **(D)** LO2 cells were treated with puerarin at the indicated concentrations for 24 h and then treated in culture medium containing TNF-α (100 ng/mL) for 24 h. Cell viability was determined by MTS assay (n = 5). **(E)** LO2 cells were treated with puerarin (20 μM) for 24 h and incubated with TNF-α (100 ng/mL) for 24 h, indicated proteins were detected by Western blotting. Data were expressed as mean ± SD. *P < 0.05, **P < 0.01, ***P < 0.001.

Since TLR4 is universally expressed in most, if not all, cells, we next accessed the direct anti-sepsis effect of puerarin on hepatocytes. In LO2 (a non-tumor human liver cell line) cells, puerarin showed no significant inhibition of cell growth at the concentrations of lower than 20 μM, proved its safety to hepatocytes under this level (**Figure 5A**). Thus, the concentration of 20 μM was chosen for the further studies. In consist with our *in vivo* findings, the activation of TLR4/NF-κB pathway in LO2 cells by LPS (100 ng/ml) was significantly suppressed by puerarin (**Figure 5B**), so did LPS stimulated IL-6 transcription. Noted of, the changes of cleaved Caspase3 and p-JNK according to different treatments, LPS vs. LPS+PUE, for example, were moderate in all time points (**Figure 5B**). Similarly, the changes of TNF-α and IL-1β expressions were limited (**Figure 5C**), suggesting LPS dominantly promoted hepatocyte inflammation, but not apoptosis. These results suggested that the massive apoptosis of hepatocytes in sepsis mice was not directly mediated by LPS, but triggered by an addition-sourced apoptotic effector, such as activated macrophages that secreted TNF-α. We tested this notion by assessing the protective effect of puerarin to TNF-α challenged hepatocytes. As shown in **Figures 5D, E**, puerarin rescued TNF-α induced hepatocyte apoptosis and reversed TNF-α induced high ratio of Bax/Bcl-2, cleaved Caspase3 and NF-κB/JNK pathway activation.

### Puerarin Promoted an M2 Phenotype in LPS Challenged Macrophages *In Vitro*

**Figure 6:**
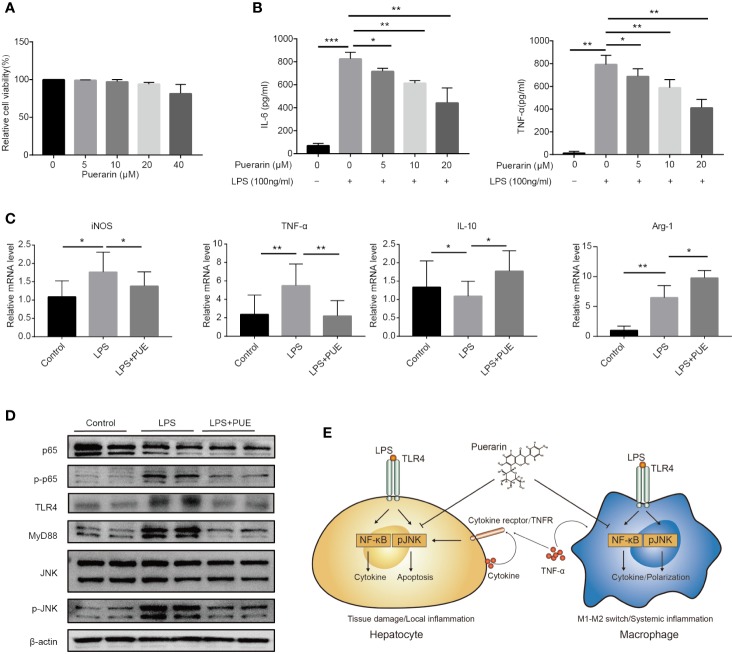
Puerarin promoted an M2 phenotype in LPS challenged macrophages *in vitro*. **(A)** RAW 264.7 cells were incubated with puerarin (20 μM) for 24 h. Cell viability was determined by MTS assay (n = 5). **(B)** The concentration of IL-6 and TNF-α in RAW 264.7 cell supernatant received LPS and puerarin treatment for 24 h were determined by ELISA (n = 4). **(C)**. The mRNA levels of iNOS, TNF-α, IL-10, and Arg-1 in the RAW 264.7 cells treated with LPS (100 ng/ml) and puerarin (20 μM) for 24 h were detected by real-time quantitative PCR (n = 4). **(D)** Western blot analyses of TLR4/Myd88/JNK/NF-κB pathway in RAW 264.7 macrophage cells treated with LPS (100 ng/ml) and puerarin (20 μM) for 24 h. **(E)** Proposed mechanism of the cellular function of puerarin and the cross talk between hepatocytes and macrophages in sepsis was illustrated. In hepatocytes, LPS dominantly induced NF-κB with limited effect on JNK activation, causing a hepatocyte inflammation. The local inflammatory environment recruited macrophages in *in vivo* context. In macrophages, LPS strongly induced NF-κB and JNK activation, causing the production of large amount of inflammatory factors and M1 phenotype. Puerarin disrupted this effect by inhibiting NF-κB and JNK pathway in both hepatocytes and macrophages. Data were expressed as mean ± SD, *P < 0.05, **P < 0.01, ***P < 0.001.

To access the direct effect of puerarin on macrophages, a commonly used macrophage cell line RAW264.7 was adopted. While puerarin showed no obvious effect on viability in these cells (**Figure 6A**), it inhibited LPS (100 ng/ml) induced release of proinflammatory cytokine IL-6 and TNF-α in a dose-dependent manner (**Figure 6B**). Importantly, puerarin decreased the mRNA levels of macrophage 1 (M1) marker iNOS and TNF-α, and increased M2 marker IL-10 and Arg-1 (Davis et al., 2013) in LPS challenged RAW264.7 cells (**Figure 6C**), suggesting a function of puerarin in polarizing macrophages from a pro-inflammation to an anti-inflammation phenotype. Similarly, as showed in the hepatocytes, puerarin suppressed the activation of TLR4/NF-κB/JNK signaling pathways in LPS treated macrophages by downregulating the phosphorylation levels of p65 and JNK. In consistent, the protein levels of TLR4 and its key mediator MyD88 were also downregulated (**Figure 6D**). In conclusion, puerarin exhibited a strong anti-sepsis effect by preventing cell death in organs and by promoting anti-inflammatory M2 differentiation in macrophages, likely *via* the suppression of TLR4/NF-κB/JNK pathways (**Figure 6E**).

## Discussion

Sepsis represents a life-threatening condition involving multiple-organ dysfunctions that result from dysregulated inflammation and immune response against infection, lacking proper treatment (Rice and Bernard, 2005). In preclinical studies, both CLP (Rittirsch et al., 2009) and LPS were broadly used in sepsis researches (Buras et al., 2005). Here, we found no obvious differences in the disease characteristics between the two models at histopathological and molecular levels. And more importantly, we showed that puerarin remarkably increased survival, suppressed systemic inflammation and prevented multiple-organ injury in both models, confirming the major contribution of LPS in, at least, gut-bacteria-originated sepsis development. Thus, LPS model was chosen as the primary model in our further experiments.

During sepsis, liver is the most vulnerable organ among the various ones to be injured (Yan et al., 2014) and its injury is one of the most life-threatening pathological events as it plays a central role in detoxifying endotoxin and producing inflammatory factors (Strnad et al., 2017). Thus, the protection of liver function is critical in sepsis treatment. It was suggested that the protective effect of puerarin on the liver in metabolic diseases, e.g., in T2DM and chronic alcohol induced liver damage, was mainly owing to its strong anti-oxidative function (Yang et al., 2009; Zhao et al., 2016). However, unlike in the metabolic diseases, as well as in lead (Liu et al., 2012) or carbon tetrachloride (CCL_4_) (Li et al., 2013b) induced liver injury, LPS-induced acute and strong liver injury appeared no significant alternation of systemic oxidant condition in mice, judged by the detection of the system oxidative stress markers, SOD and MDA, in the serum in current study. Instead, we observed an effective and direct inhibition of inflammatory signaling in macrophages and hepatocytes and a reverse of sepsis phenotype. Thus, evidence supported the notion that puerarin possesses both anti-oxidative and anti-inflammatory functions. Exactly why and how puerarin exhibits such a dual function upon the contexts is not clear, presumably partially due to different experimental settings that target distinct cellular processes.

Mechanistically, puerarin has been suggested to exert an anti-inflammatory effect by suppressing TLR2/NF-κB/JNK signaling in staphylococcus aureus-induced mastitis involving TLR2 (Wu et al., 2016). It prevented LPS-induced acute lung injury (Wang et al., 2018) and mediated hepatoprotection against alcohol-induced liver injury involving the suppression of NF-κB (Li et al., 2013a). However, its protective effect on TNF-α/JNK-induced hepatocyte apoptosis and on liver injury in sepsis by inhibiting TLR4/NF-κB/JNK pathways was not fully addressed.

Importantly, we observed that the direct induction of cell death and activation of JNK signaling by LPS on hepatocytes were rather minor, raising the possibility that the severe *in vivo* liver cell death caused by LPS was triggered by additional factors such as TNF-α that is known to be produced mainly by macrophages which was much enriched in the livers in sepsis (**Figure 3B**). TNF-α is a highly pleiotropic cytokine, inducing various biological processes, including cell proliferation, metabolic activation, inflammatory responses, and apoptotic/necrotic cell death, depending on the context that the cells engaged (Liedtke and Trautwein, 2012). In sepsis, macrophages that exposed to living bacteria or LPS produced large amount of TNF-α, causing massive cell death, organ injury, and fatal shock syndromes that developed in sepsis (Pfeffer et al., 1993), apparently *via* the over activation of JNK signaling by TNF-α as mice that lack of JNK gene are protected from TNF-α-induced hepatotoxicity (Kodama et al., 2009).

Noted of, NF-κB activation itself induced by LPS at cellular level promotes gene expressions with potentially antiapoptotic functions, including the expressions of cellular inhibitors of apoptosis (cIAPs) and antiapoptotic Bcl2 family members (Karin and Lin, 2002). It also triggers a cellular inflammatory response by expressing mainly the proinflammatory signal IL-6 that itself is also pro-survival (Tominaga et al., 1997), while initiating an appropriate immune action in the liver (Luedde and Trautwein, 2006). It is the uncontrolled and prolonged JNK activation in the liver presumably upon a massive activation and invasion of LPS “hijacked” macrophages, which caused severe hepatocyte apoptosis (Das et al., 2009) by expressing high level of TNF-α.

Previously, it is believed that in sepsis, the immune homeostasis imbalance, mainly manifested as cytokine storm and lymphocyte apoptosis and malfunction (Hotchkiss and Karl, 2003) is crucial in sepsis-mediated host death (Rittirsch et al., 2008). Cytokine storm is an important pathological state in the early stage of sepsis causing capillary leak syndrome and multiple-organ failures (Tisoncik et al., 2012). Macrophages play a key role in regulating the balance between effective immune response and hyperactivation of inflammation (Odegaard and Chawla, 2011). In sepsis, endotoxin stimulates the synthesis and release of various pro-inflammatory cytokines and factors, including TNFα, IL-1β, IL-6, Fas ligand (FasL), granzymes, etc., mainly by mononuclear macrophages. These pro-inflammatory factors accelerate the apoptosis of lymphocytes, especially CD4^+^T cells, and dendritic cells, thereby aggravating sepsis immunosuppression (Hotchkiss et al., 2013). The underling mechanism is the over activation of NF-κB and JNK pathways (Himes et al., 2006; Hoesel and Schmid, 2013), which promotes macrophages' M1 transformation and systemic inflammation (Han et al., 2013). On this view, puerarin effectively reversed the macrophage M1 activation by LPS through the inhibition of TLR4/NF-κB/JNK pathways and promoted a M2 phenotype, which contributed to cease a cytokine storm. However, the function of puerarin in increasing the proportion of functional DCs and T helper cells was limited, suggesting that while to initiate an appropriate immune action may be important in preventing repeated infections, our evidence argue that the recovery of T-dependent immune responses was not necessary on life-saving in early stage of sepsis. Thus, an effective blockage of macrophage activation by suppressing its TLR4/NF-κB/JNK pathways at early phage of sepsis is crucial.

Together, puerarin protected against sepsis through two main cellular effects. It directly suppresses cellular inflammatory response in somatic cells, such as hepatocytes, in organs locally, and it targets macrophages to prevent an over activation of inflammation systemically, mainly through the inhibition of TLR4/NF-κB/JNK pathways. Importantly, this study suggested puerarin's therapeutic potential in sepsis.

## Data Availability Statement

The datasets generated for this study are available on request to the corresponding authors.

## Ethics Statement

The animal study was reviewed and approved by Committee of the Care and Use of Laboratory Animals (Nanjing University).

## Author Contributions

LW and QL carried out the histological analysis, the animal experiment and drafted the manuscript. AL and XC carried out the cell culture and flow cytometric analysis. YzW and BZ participated in the animal experiment. YZ and HM participated in the western blotting, qPCR, and ELISA. YtW participated in the design of the study and performed the statistical analysis. SS and QG conceived of the study, and participated in its design and coordination and helped to draft the manuscript. All authors read and approved the final manuscript.

## Conflict of Interest

The authors declare that the research was conducted in the absence of any commercial or financial relationships that could be construed as a potential conflict of interest.

## References

[B1] Angus D. C. van der Poll T. (2013). Severe Sepsis and Septic Shock. New Engl. J. Med. 369, 840–851. 10.1056/NEJMra1208623 23984731

[B2] Ascon M. Ascon D. B. Liu M. Cheadle C. Sarkar C. Racusen L. (2009). Renal ischemia-reperfusion leads to long term infiltration of activated and effector-memory T lymphocytes. Kidney Int. 75, 526–535. 10.1038/ki.2008.602 19092796 PMC2676145

[B3] Ashare A. Monick M. M. Powers L. S. Yarovinsky T. Hunninghake G. W. (2006). Severe bacteremia results in a loss of hepatic bacterial clearance. Am. J. Resp. Crit. Care 173, 644–652. 10.1164/rccm.200509-1470OC PMC2662948 16399991

[B4] Baranova I. N. Souza A. C. Bocharov A. V. Vishnyakova T. G. Hu X. Vaisman B. L. (2016). Human SR-BI and SR-BII Potentiate Lipopolysaccharide-Induced Inflammation and Acute Liver and Kidney Injury in Mice. J. Immunol. 196, 3135–3147. 10.4049/jimmunol.1501709 26936883 PMC4856165

[B5] Buras J. A. Holzmann B. Sitkovsky M. (2005). Animal models of sepsis: Setting the stage. Nat. Rev. Drug Discovery 4, 854–865. 10.1038/nrd1854 16224456

[B6] Canabal J. M. Kramer D. J. (2008). Management of sepsis in patients with liver failure. Curr. Opin. Crit. Care 14, 189–197. 10.1097/MCC.0b013e3282f6a435 18388682

[B7] Das M. Sabio G. Jiang F. Rincon M. Flavell R. A. Davis R. J. (2009). Induction of Hepatitis by JNK-Mediated Expression of TNF-alpha. Cell 136, 249–260. 10.1016/j.cell.2008.11.017 19167327 PMC2794880

[B8] Davis M. J. Tsang T. M. Qiu Y. Dayrit J. K. Freij J. B. Huffnagle G. B. (2013). Macrophage M1/M2 Polarization Dynamically Adapts to Changes in Cytokine Microenvironments in Cryptococcus neoformans Infection. mBio 4, e002684–13. 10.1128/mBio.00264-13 PMC3684832 23781069

[B9] Han M. S. Jung D. Y. Morel C. Lakhani S. A. Kim J. K. Flavell R. A. (2013). JNK Expression by Macrophages Promotes Obesity-Induced Insulin Resistance and Inflammation. Science 339, 218–222. 10.1126/science.1227568 23223452 PMC3835653

[B10] Himes S. R. Sester D. P. Ravasi T. Cronau S. L. Sasmono T. Hume D. A. (2006). The JNK are important for development and survival of macrophages. J. Immunol. 176, 2219–2228. 10.4049/jimmunol.176.4.2219 16455978

[B11] Hoesel B. Schmid J. A. (2013). The complexity of NF-kappa B signaling in inflammation and cancer. Mol. Cancer 12, 86. 10.1186/1476-4598-12-86 23915189 PMC3750319

[B12] Hotchkiss R. S. Karl I. E. (2003). Medical progress: The pathophysiology and treatment of sepsis. New Engl. J. Med. 348, 138–150. 10.1056/NEJMra021333 12519925

[B13] Hotchkiss R. S. Monneret G. Payen D. (2013). Sepsis-induced immunosuppression: from cellular dysfunctions to immunotherapy. Nat. Rev. Immunol. 13, 862–874. 10.1038/nri3552 24232462 PMC4077177

[B14] Hu W. Yang X. Zhe C. Zhang Q. Sun L. Cao K. (2011). Puerarin inhibits iNOS, COX-2 and CRP expression via suppression of NF-kappaB activation in LPS-induced RAW264.7 macrophage cells. Pharmacol. Rep. 63, 781–789. 10.1016/S1734-1140(11)70590-4 21857089

[B15] Iskander K. N. Osuchowski M. F. Stearns-Kurosawa D. J. Kurosawa S. Stepien D. Valentine C. (2013). Sepsis: Multiple Abnormalities, Heterogeneous Responses, And Evolving Understanding. Physiol. Rev. 93, 1247–1288. 10.1152/physrev.00037.2012 23899564 PMC3962548

[B16] Karin M. Lin A. (2002). NF-kappaB at the crossroads of life and death. Nat. Immunol. 3, 221–227. 10.1038/ni0302-221 11875461

[B17] Kodama Y. Taura K. Miura K. Schnabl B. Osawa Y. Brenner D. A. (2009). Antiapoptotic effect of c-Jun N-terminal Kinase-1 through Mcl-1 stabilization in TNF-induced hepatocyte apoptosis. Gastroenterology 136, 1423–1434. 10.1053/j.gastro.2008.12.064 19249395

[B18] Kramer L. Jordan B. Druml W. Bauer P. Metnitz P. G. H. (2007). Incidence and prognosis of early hepatic dysfunction in critically ill patients - A prospective multicenter study. Crit. Care Med. 35, 1099–1104. 10.1097/01.CCM.0000259462.97164.A0 17334250

[B19] Li R. Liang T. He Q. Guo C. Xu L. Zhang K. (2013a). Puerarin, isolated from Kudzu root (Willd.), attenuates hepatocellular cytotoxicity and regulates the GSK-3beta/NF-kappaB pathway for exerting the hepatoprotection against chronic alcohol-induced liver injury in rats. Int. Immunopharmacol. 17, 71–78. 10.1016/j.intimp.2013.05.023 23751897

[B20] Li R. Xu L. Liang T. Li Y. Zhang S. Duan X. (2013b). Puerarin mediates hepatoprotection against CCl4-induced hepatic fibrosis rats through attenuation of inflammation response and amelioration of metabolic function. Food Chem. Toxicol. 52, 69–75. 10.1016/j.fct.2012.10.059 23146695

[B21] Liedtke C. Trautwein C. (2012). The role of TNF and Fas dependent signaling in animal models of inflammatory liver injury and liver cancer. Eur. J. Cell Biol. 91, 582–589. 10.1016/j.ejcb.2011.10.001 22153863

[B22] Liu C. Ma J. Sun Y. (2012). Puerarin protects the rat liver against oxidative stress-mediated DNA damage and apoptosis induced by lead. Exp. Toxicol. Pathol. 64, 575–582. 10.1016/j.etp.2010.11.016 21146379

[B23] Luedde T. Trautwein C. (2006). Intracellular survival pathways in the liver. Liver Int. 26, 1163–1174. 10.1111/j.1478-3231.2006.01366.x 17105581

[B24] Martin G. S. Mannino D. M. Eaton S. Moss M. (2003). The epidemiology of sepsis in the United States from 1979 through 2000. New Engl. J. Med. 348, 1546–1554. 10.1056/NEJMoa022139 12700374

[B25] Matute-Bello G. Downey G. Moore B. B. Groshong S. D. Matthay M. A. Slutsky A. S. (2011). An Official American Thoracic Society Workshop Report: Features and Measurements of Experimental Acute Lung Injury in Animals. Am. J. Resp. Cell Mol. 44, 725–738. 10.1165/rcmb.2009-0210ST PMC7328339 21531958

[B26] Odegaard J. I. Chawla A. (2011). “Alternative Macrophage Activation and Metabolism,” in Annual Review of Pathology-Mechanisms of Disease. Ed. Abbas G Howley A. K. S. J. P. M. (PALO ALTO: ANNUAL REVIEWS), 275–297. 10.1146/annurev-pathol-011110-130138 PMC3381938 21034223

[B27] Pfeffer K. Matsuyama T. Kundig T. M. Wakeham A. Kishihara K. Shahinian A. (1993). Mice deficient for the 55 kd tumor necrosis factor receptor are resistant to endotoxic shock, yet succumb to L. monocytogenes infection. Cell 73, 457–467. 10.1016/0092-8674(93)90134-c 8387893

[B28] Rice T. W. Bernard G. R. (2005). Therapeutic intervention and targets for sepsis. Annu. Rev. Med. 56, 225–22+. 10.1146/annurev.med.56.082103.104356 15660511

[B29] Rittirsch D. Flierl M. A. Ward P. A. (2008). Harmful molecular mechanisms in sepsis. Nat. Rev. Immunol. 8, 776–787. 10.1038/nri2402 18802444 PMC2786961

[B30] Rittirsch D. Huber-Lang M. S. Flierl M. A. Ward P. A. (2009). Immunodesign of experimental sepsis by cecal ligation and puncture. Nat. Protoc. 4, 31–36. 10.1038/nprot.2008.214 19131954 PMC2754226

[B31] Russell J. A. (2006). Drug therapy: Management of sepsis. New Engl. J. Med. 355, 1699–1713. 10.1056/NEJMra043632 17050894

[B32] Schindelin J. Arganda-Carreras I. Frise E. Kaynig V. Longair M. Pietzsch T. (2012). Fiji: an open-source platform for biological-image analysis. Nat. Methods 9, 676–682. 10.1038/nmeth.2019 22743772 PMC3855844

[B33] Shapiro N. I. Howell M. D. Talmor D. Donnino M. Ngo L. Bates D. W. (2007). Mortality in Emergency Department Sepsis (MEDS) score predicts 1-year mortality. Crit. Care Med. 35, 192–198. 10.1097/01.CCM.0000251508.12555.3E 17110874

[B34] Strnad P. Tacke F. Koch A. Trautwein C. (2017). Liver - guardian, modifier and target of sepsis. Nat. Rev. Gastro. Hepat. 14, 55–66. 10.1038/nrgastro.2016.168 27924081

[B35] Tisoncik J. R. Korth M. J. Simmons C. P. Farrar J. Martin T. R. Katze M. G. (2012). Into the Eye of the Cytokine Storm. Microbiol. Mol. Biol. R. 76, 16–32. 10.1128/MMBR.05015-11 PMC3294426 22390970

[B36] Tominaga K. Kirikae T. Nakano M. (1997). Lipopolysaccharide (LPS)-induced IL-6 production by embryonic fibroblasts isolated and cloned from LPS-responsive and LPS-hyporesponsive mice. Mol. Immunol. 34, 1147–1156. 10.1016/s0161-5890(97)00145-4 9566762

[B37] Wang X. Yan J. Xu X. Duan C. Xie Z. Su Z. (2018). Puerarin prevents LPS-induced acute lung injury via inhibiting inflammatory response. Microb. Pathog. 118, 170–176. 10.1016/j.micpath.2018.03.033 29571724

[B38] Wenzel R. P. Edmond M. B. (2015). Antibiotics for Abdominal Sepsis. New Engl. J. Med. 372, 2062–2063. 10.1056/NEJMe1503936 25992751

[B39] Wong K. H. Li G. Q. Li K. M. Razmovski-Naumovski V. Chan K. (2011). Kudzu root: Traditional uses and potential medicinal benefits in diabetes and cardiovascular diseases. J. Ethnopharmacol. 134, 584–607. 10.1016/j.jep.2011.02.001 21315814

[B40] Wu H. Zhao G. Jiang K. Chen X. Zhu Z. Qiu C. (2016). Puerarin Exerts an Antiinflammatory Effect by Inhibiting NF-kB and MAPK Activation in Staphylococcus aureus-Induced Mastitis. Phytother. Res. 30, 1658–1664. 10.1002/ptr.5666 27335240

[B41] Yan J. Li S. Li S. (2014). The Role of the Liver in Sepsis. Int. Rev. Immunol. 33, 498–510. 10.3109/08830185.2014.889129 24611785 PMC4160418

[B42] Yang S. Lou J. L. Wang Q. (2009). Effect of puerarin on liver injury in KKAy mice with type 2 diabetes mellitus. Zhongguo Zhong Xi Yi Jie He Za Zhi 29, 707–710. 19848202

[B43] Zhang Z. Lam T. Zuo Z. (2013). Radix Puerariae: An overview of Its Chemistry, Pharmacology, Pharmacokinetics, and Clinical Use. J. Clin. Pharmacol. 53, 787–811. 10.1002/jcph.96 23677886

[B44] Zhao L. Wang Y. Liu J. Wang K. Guo X. Ji B. (2016). Protective Effects of Genistein and Puerarin against Chronic Alcohol-Induced Liver Injury in Mice via Antioxidant, Anti-inflammatory, and Anti-apoptotic Mechanisms. J. Agric. Food Chem. 64, 7291–7297. 10.1021/acs.jafc.6b02907 27609057

[B45] Zhou Y. Zhang H. Peng C. (2014). Puerarin: A Review of Pharmacological Effects. Phytother. Res. 28, 961–975. 10.1002/ptr.5083 24339367

